# Oral Manifestations of Graft vs. Host Disease: A Comprehensive Review for Best Practice in Dentistry

**DOI:** 10.3390/medicina59111937

**Published:** 2023-11-01

**Authors:** Michele Miranda, Francesco Gianfreda, Danesi Carlotta, Sofia Armati, Alberta Barlattani, Patrizio Bollero

**Affiliations:** 1 Department of Clinical Sciences and Translational Medicine, University of Rome “Tor Vergata”, 00133 Rome, Italy; 2 Department of Industrial Engineering, University of Rome “Tor Vergata”, 00133 Rome, Italy; 3 Private Practice, 20122 Milan, Italy; 4 Department of System Medicine, University of Rome “Tor Vergata”, 00133 Rome, Italy

**Keywords:** graft-versus-host disease, oral manifestations, dental treatment

## Abstract

Graft-versus-host disease (GVHD) is a complication of hematopoietic stem cell transplantation (HSCT). GVHD may also develop following solid transplants or blood transfusions if white blood cells are transferred. GVHD affects multiple organs, including the oral tissues. This pictorial review provides a background of GVHD to dental practitioners, describes the most common oral manifestations of GVHD, and highlights the main treatment modifications needed to deliver dental care to patients with GVHD. A narrative review enriched with clinical data was performed by searching the scientific literature for all articles regarding GVHD and oral manifestations/therapies. All articles without exclusion criteria, except animal tests, were included in the above review. Acute GVHD may manifest in the oral mucosa; however, it often develops immediately following HSCT when routine dental treatment is postponed. Chronic GVHD may manifest in the oral mucosa, the salivary glands, and the musculoskeletal compartment. It may indirectly affect the teeth and the oral flora, putting the patient at risk for infections. Importantly, GVHD poses an increased risk for oral cancer. GVHD has a wide range of oral manifestations, some of which may affect dental treatment.

## 1. Introduction

GvHD is known as the worst complication of allogeneic hematopoietic stem cell (allo-HSCT) transplants, making it the leading cause of morbidity and mortality associated with this transplant [1]. The immunological mechanism underlying GvHD can be considered as “opposite” to rejection: while in the latter case, the host’s immune cells recognize the cells of the transplanted tissue as “non-self”, in the graft-versus-host disease it is the transplanted immune cells that recognize the recipient’s organs as foreign and trigger an immune attack against them [2,3].

In 1960, Billingham established the criteria for the onset of GvHD:

1. Graft containing supply of immunocompetent cells [4]
2. Immunological dissimilarity between host and donor
3. Immunosuppressed host.

### 1.1. How It Works: Allo-HSCT

The Billingham criteria can be found in allo-HSCT, a transplant that consists of the infusion of hematopoietic stem cells from a compatible donor into a recipient who has been appropriately conditioned with high-dose chemo- and radiotherapy [2]. The goals of chemo-radiotherapy treatment and the infusion of allogeneic hematopoietic stem cells are:

- Resolution of the disease
- Allogeneic stem cell engraftment (production of the necessary space for the infusion)
- Bone marrow restoration by the infused cells after a period of aplasia (which takes about 100 days)
- Elimination of remaining diseased cells thanks to the graft-versus-tumor effect (GvT): allogeneically transplanted human hematopoietic cells can eliminate residual tumor cells in the recipient by an immune-mediated mechanism [3].

The main indication for this transplant is acute myeloid leukemia, but it is not the only one [5].

The compatibility of HLA genes must be the same (autotransplant) or very similar since the reduction in compatibility is associated with an increasing risk of developing GvHD [4,6]. Technological advancement and a better understanding of the characteristics of the epitope structure of HLA antigens have allowed the development of a test that identifies the compatibility between patient and possible donor, which is achieved through a tool, the HLA-MatchMaker, a computerized algorithm that analyzes a simple venous blood draw from both the donor and the recipient [7,8]. The probability of finding a compatible donor in the family environment, given the huge polymorphism of the HLA system, is around 25%, while the probability of finding it in the National and International Registers of bone marrow donors is around 40% [9].

The sources of stem cells used can be:

- Bone marrow (BM).
- Peripheral blood stem cells (PBSC), which is the most innovative and most used technique to date.
- Umbilical cord blood, which, however, does not provide a quantity of stem cells sufficient to treat an adult.

### 1.2. Pre-HSCT Dentistry Videat

In patients who must undergo HSCT, the consensus is that all low-invasive dental care is necessary to eliminate any traumatic factors and infectious foci, postponing any elective treatments (such as extended surgical treatments) after the post-transplant immune reconstitution [10]. The trend is to end the necessary dental therapies about 10 days before the transplant, to give the patient time to undergo conditioning therapy [11]. The current protocol, based on the most frequently encountered pathologies, is summarized in Table 1 [4].

Traumatic factors (overflowing restorations, fractured teeth, root debris) could be etiological factors in the development of oral ulcers or contribute to the development of oral mucositis in HSCT transplant patients who develop GvHD. Therefore, these should be eliminated [12]. For the same reason, prostheses should be cleaned and relined to perfection. No dental therapy should ever be performed in the 6–12-month post-transplant period, including dental hygiene maneuvers that could generate aerosols with accidental aspiration of bacteria or debris [13].

#### Epidemiology

Approximately 35–50% of patients undergoing HSCT develop acute GvHD. In addition, it has been estimated that about 50% of patients with the acute form also subsequently develop the chronic form [14].

Regarding prevalence, a 2018 study by the U.S. Research and Markets statistical center calculated that in the seven largest pharmaceutical markets, diagnosed cases of chronic and acute GvHD stood at around 18,408, set to rise above 22,000 in 2028.

### 1.3. Acute and Chronic GVHD

In the past, GvHD was divided into two main forms: acute and chronic. This designation was based exclusively on a temporal criterion, the so-called “10-day dividing line”. The 2005 NIH Consensus Conference abolished this classification criterion and redefined chronic and acute GvHD solely based on different clinical manifestations [15]. Acute GvHD (aGvHD) is thus subdivided into classic and late forms, based on whether symptoms occur before or after 100 days post-transplant. The late form is further subdivided into the following forms:

- Persistent, if characterized by the persistence of signs and symptoms of classic aGvHD initiated before day 100 post-transplant;
- Recurrent, if it arises as a classic form that resolves but recurs after day 100 post-transplant;
- De novo, if it occurs after day 100 post-transplant without any signs or symptoms in the previous time frame.

As far as the chronic form (cGvHD) is concerned, this can arise following the evolution of the acute form (PTO, progressive type of onset), after the resolution of a previous acute form (quiescent), or “de novo”. Also, cGvHD is divided into classic and superimposed forms, if it occurs with the typical acute form manifestations on a pre-existing chronic form [16].

#### 1.3.1. aGvHD Pathogenesis

The pathogenesis of acute GvHD can be divided into three phases:

- Afferent phase: this concerns the phase prior to HSC transplantation, i.e., conditioning. The use of high-dose chemotherapy and radiotherapy induces tissue damage in the patient, with the secretion of numerous pro-inflammatory cytokines such as IL-1 and TNF-alpha. These will lead to an overexpression of adhesion molecules and antigens on MHCs. This event will facilitate the recognition of these cells as non-self by donor immune cells once transplantation has taken place [17].
- Induction and expansion phase: after transplantation, the recognition of host antigens by donor T cells, which, once activated, will undergo proliferation and differentiation [18]. The contact of CD4+ cells with class II MCHs and CD8+ cells with class I MCHs will cause the production of several cytokines with a decisive role in the development of the disease, such as IL-2 and IFN-gamma. Under the effect of these cytokines, lymphocytes become active and proliferate and T-helper lymphocytes will be prompted to take the differentiation pathway that will lead them to present more of a Th1 phenotype [19].
- Effector phase: after their activation in the previous phase, T cells secrete a disproportionate number of cytokines that lead to migration, activation, and proliferation of other immune cells. This phenomenon, due to the high production of cytokines, is called cytokine storm [20]. Cytokine activity, however, only partially explains GvHD damage. Although these play a primary role in the morbidity and mortality of systemic GvHD, they are less important in damaging specific target organs of this disease, which are the gut, skin, and liver. Thus, the effector phase will be characterized by cytotoxic damage caused by activated donor CD8+ T cells (further invoked by the action of Th1 lymphocytes) against host cells of the target organs [19,21].

#### 1.3.2. cGvHD Pathogenesis

Despite the important progress in recent years in understanding the pathogenetic mechanisms underlying aGvHD, chronic GvHD is much less defined. Based on clinical features, chronic GvHD is now considered an autoimmune phenomenon in which tolerance for host tissues is lost and is characterized by the production of autoantibodies (mainly IgM and IgG), monoclonal gammopathies, and numerous connective tissue sclerotic phenomena. In the development of cGvHD, we have the failure of the response that involves mainly CD8+ and CD4+ lymphocytes with a lack of production of INF-gamma and IL-2, which are the cytokines characterizing acute GvHD and allow the selection of the Th1 phenotype [22]. The production of cytokines allows the preferential selection of the Th2 phenotype and the consequent hyperactivation of B cells with the production of autoantibodies that characterize the chronic form of the disease [23]. The reasons behind the failure of this response and the altered secretion of interferon-gamma and IL-2, which will subsequently lead to the selection of the Th2 phenotype in cGvHD, still remain a question to be solved [24]. Many cytokines and chemokines are involved in the pathogenetic process and mediate tissue damage of target organs in this form of autoimmune disease:

- TGF-beta is the cytokine involved in the stimulation of collagen synthesis in the sclerosis phenomena characteristic of skin and mucous membranes in this disease;
- IL-4 stimulates the production of IgG and IgE by B cells, but also the proliferation of mast cells that play a primary role in the skin alterations typical of cGvHD;
- IL-5 is correlated with an increase in circulating eosinophils;
- IL-10: the association of IL-10 and high concentrations of TGF-β, is associated with immunodeficiency.

#### 1.3.3. aGvHD Clinical Manifestations

The clinical manifestations of aGvHD, the expression of an immune deficiency, mainly concern:

1. Skin involvement (81%) is characterized by the presence of a maculo-papular, itchy, violet-colored rash. The distribution of the rash is characteristic: at first, it involves the palms of the hands and feet soles, then it spreads to the cheeks, ears, neck, trunk, chest, and back. Histological findings show apoptotic phenomena of cells of the deep layers at the level of epithelial ridges, invaginations of epithelium that deepen in the dermis, with dermal–epidermal separation. Other typical findings are dyskeratosis, perivascular lymphocytic infiltration at the level of the dermis, and the presence of satellite lymphocytes in close proximity to dyskeratotic keratinocytes [25].
2. Gastro-intestinal involvement (54%), where the most common symptom is diarrhea, but emesis, anorexia, abdominal pain, or a combination of painful cramps, occasional bleeding, and ileus (intestinal occlusion) may also occur in severe cases. Histologically present are flattening of the intestinal villi, dilation of the intestinal lumen and thickening of the walls of the small intestine, inflammation of the lamina propria, destruction of the crypts, lesions ranging from unicellular necrosis of the mucosal epithelium to complete loss of the epithelium itself and possible ulcerations surrounded by erythematous halos [22].
3. Hepatic involvement (50%), manifested by cholestatic hepatopathy, with or without jaundice, and hepatomegaly. In hematochemical examinations, direct bilirubin, gamma-GT, and alkaline phosphatase levels (also individually) are altered, while transaminases (GOT and GPT) show a nonspecific increase.
4. Eye involvement, with dry keratoconjunctivitis and a peculiar pseudomembranous conjunctivitis [20,26].

#### 1.3.4. cGvHD Clinical Manifestations

The clinical manifestations of cGvHD, the expression of an autoimmune disease, mainly concern:

1. Skin (80%), in which skin lesions are characterized by initial dry skin and ichthyosis (fish-scaled skin), then progressing to an itchy, purplish, plaque or papular rash, clinically indistinguishable from lichen planus. Another typical cutaneous manifestation is superficial or deep dermal fibrosis [27,28]. There are also some erythematous areas that precede the fibrotic lesion with plaques that mainly affect the skin of the lower limbs, joints, and bones. Skin appendages are affected with focal or diffuse alopecia and nail dystrophy [28].
2. At the musculoskeletal level, fibrosis can also affect the tendons and muscles, causing fasciitis, myositis with muscle weakness, myalgia, and muscle contractures that facilitate diagnosis; in fact, muscle biopsies show phenomena of degeneration, necrosis, and regeneration of muscle fibers. Musculoskeletal system impairment seriously compromises the patient’s life quality, impacting his mobility [28].
3. Respiratory patients who see lung involvement are often asymptomatic or have nonspecific symptoms at an early stage and, as a result, diagnosis is often delayed. The patient often presents exertional dyspnea, sinusitis, reduced tolerance to physical exercise, and persistent cough at the time of diagnosis. The cause of these alterations is to be traced back to obliterating bronchiolitis, which if it continues without being subjected to therapy can lead to the death of the patient from serious superinfections [29].
4. In the liver, the alterations are like the acute form, with cholestasis and increased serum levels of bilirubin and alkaline phosphatase (ALP).
5. Ocular symptoms such as xerophthalmia and photophobia, due to keratoconjunctivitis sicca (KCS) also known as dry eye syndrome, or other types of keratopathies.
6. In women, genital involvement may be the only sign of chronic GvHD. These signs develop on average 10 months after the transplant and include dryness, erosions, thickening of the mucosa with vaginal narrowing, frequent infections, and pain during sexual activity (dyspareunia). In men, more rarely, the presence of phimosis can be highlighted [20].

### 1.4. Oral GVHD

**Figure 1:**
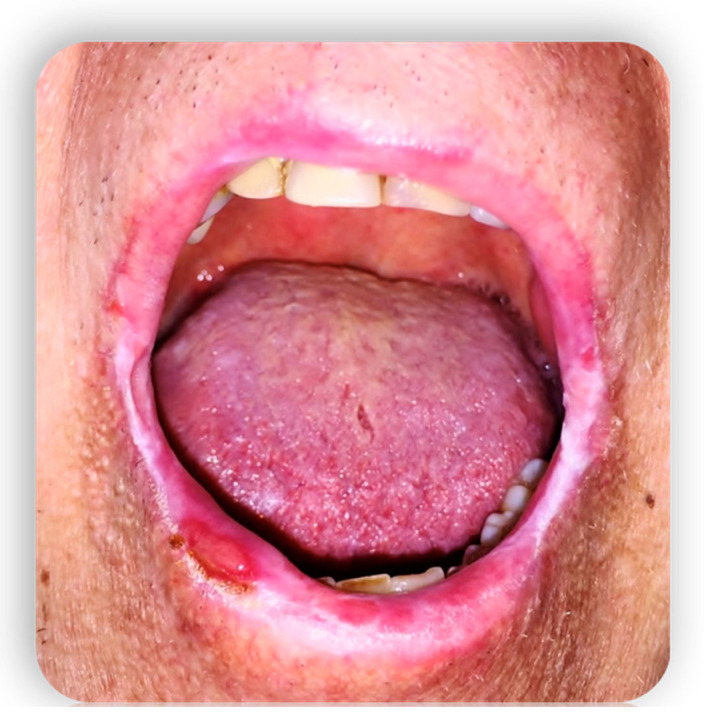
Oral mucositis manifestations from GvHD.

**Figure 2:**
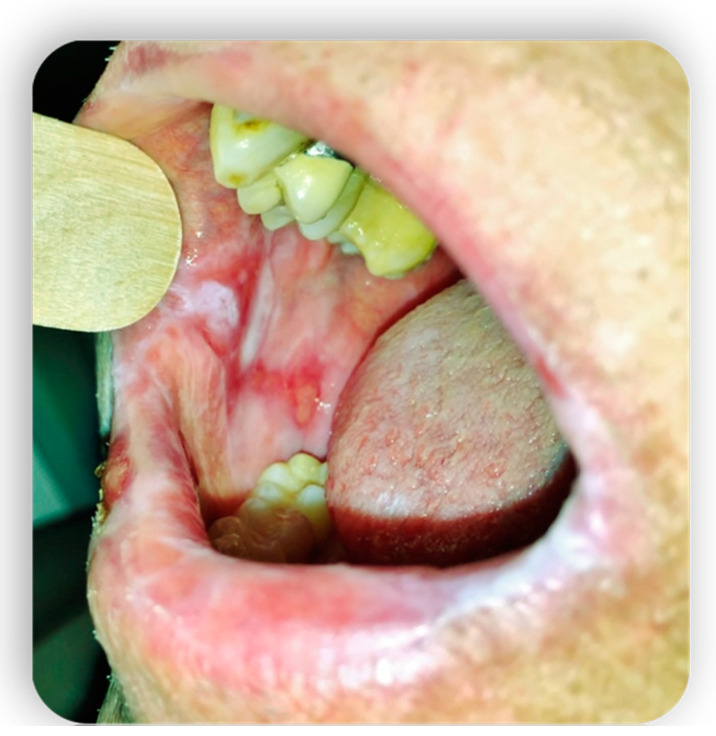
Oral mucositis manifestations from GvHD.

The oral manifestations of GvHD can be divided into three groups: the manifestations derived from the conditioning regimen and those of the acute and chronic form of GvHD (Figure 1 and Figure 2) [25].

#### 1.4.1. Oral Mucositis from Pre-HSCT Conditioning

Oral mucositis represents a severe inflammatory picture of the oral mucosa, a complication often present in patients undergoing chemotherapy and radiotherapy, the frequency of which rises to 75–99% in cases of pre-HSCT conditioning, due to the high doses used [23].

The WHO divides oral mucositis into four degrees based on the severity of symptoms and lesions:

- Grade 1: presence of erythema with slight discomfort, which is the initial state of the pathology.
- Grade 2: diffuse erythema and superficial ulcers that still allow solid nutrition.
- Grade 3: painful ulcers that force the patient to switch to an exclusively liquid diet due to the high chewing pain.
- Grade 4: extensive mucositis that requires parenteral nutrition is the most severe.

The maintenance of excellent oral hygiene is essential for prevention, especially to minimize the risk of secondary fungal or bacterial infections. The guidelines of the Multinational Association of Supportive Care in Cancer (MASCC) and the International Society of Oral Oncology (ISOO) recommend the use of soft brushes, dental floss, and the use of saline or sodium bicarbonate rinses. All traumatic factors can contribute to the development of inflammation of the oral mucosa and must therefore be removed [8].

Other methods to prevent the development of oral mucositis are the use of low-level laser therapy that improves tissue repair and reduces pain, zinc supplements (antioxidant), and recombinant human KGF. Analgesia in the treatment of mucositis is obtained with the use of mouthwashes containing 2% morphine, benzydamine (a local analgesic and anti-inflammatory/), or 0.5% doxepin [30].

#### 1.4.2. Oral aGvHD Manifestation

**Figure 3:**
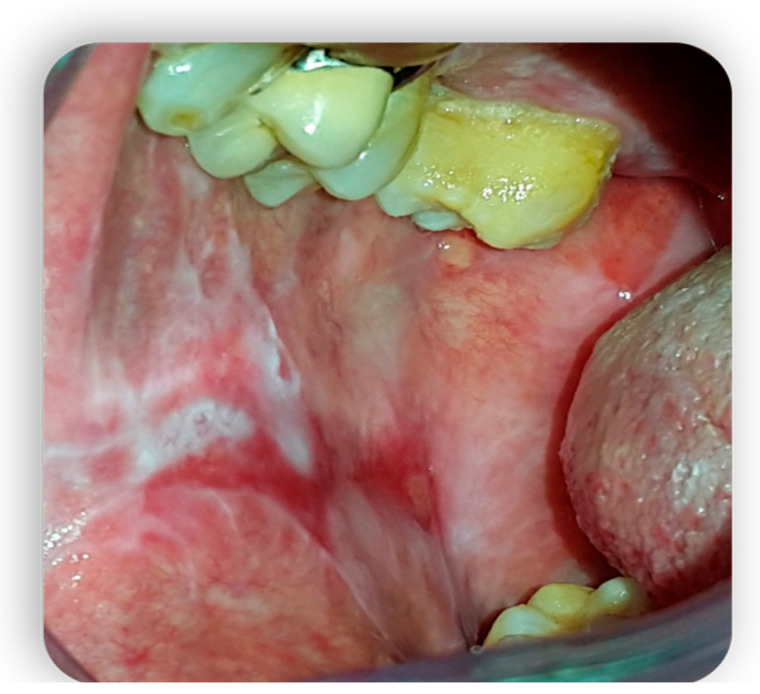
Oral mucositis manifestations from Gvhd.

**Figure 4:**
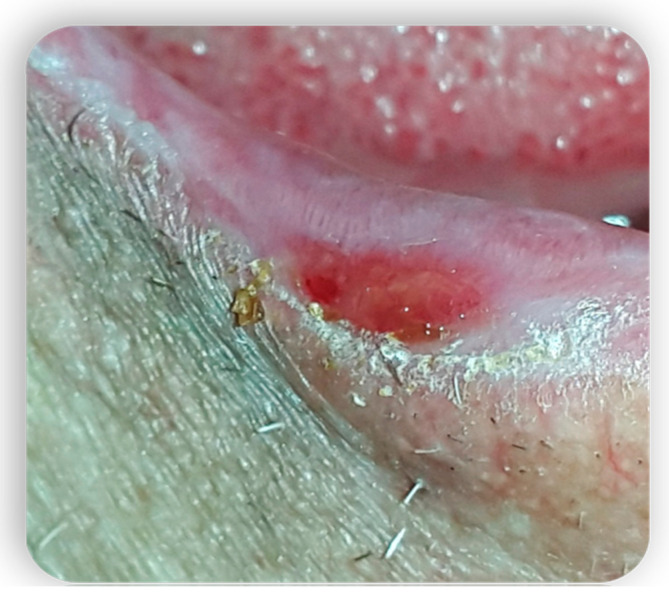
Oral mucositis manifestations from Gvhd.

The oral manifestations in acute GvHD are very infrequent and therefore there is no well-defined characterization in the literature. In fact, some authors such as Zeiser [7] in their 2004 and 2006 studies, respectively, do not consider acute oral GvHD to be a distinct clinical entity since the lesions are indistinguishable from those of conditioning. However, Ion’s work in 2014 showed lesions such as generalized erythema and pseudomembranous ulcerations [31], mucosal atrophy, and hyperkeratotic striae (lesions similar to the Wickham striae of lichen planus) [32]. Acute GvHD oral lesions are considered those that occur 3 or 4 weeks after transplantation. The sites most affected by oral aGvHD are the non-keratinized oral mucosa, the tongue (especially the ventrolateral and dorsal portion), the labial mucosa, and the hard and soft palate [30]. Gingival involvement is less frequent. In severe cases, patients may also present with xerostomia and salivary gland hypofunction. Histologically, the epithelium of the mucosa shows variations in thickness and has variable alterations ranging from dyskeratosis to reactive atypia. The signs of acute or chronic inflammation of the lamina propria are practically always present (Figure 3 and Figure 4) [33].

#### 1.4.3. Oral cGvHD Manifestation

**Figure 5:**
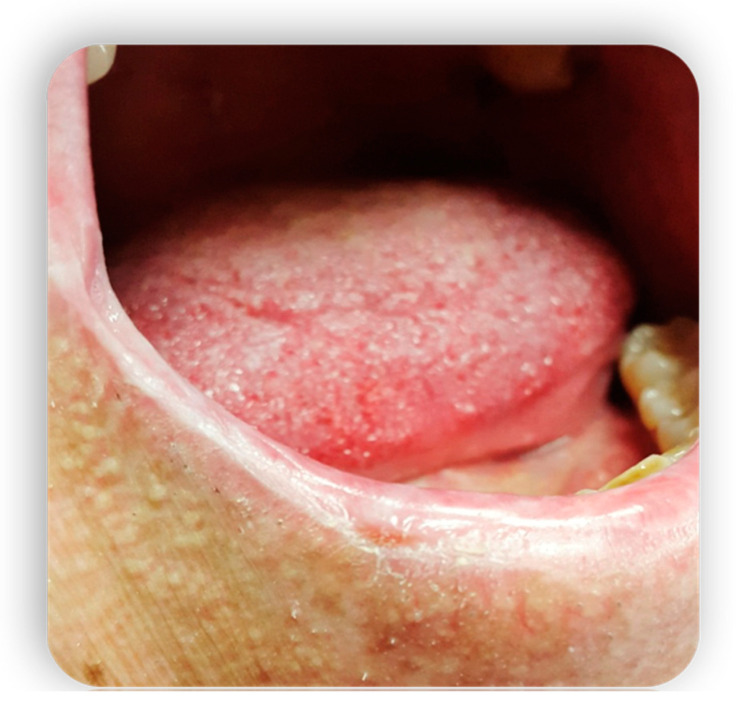
Oral cGvHD manifestation: Labial commissure ulcerations that reduce mouth opening.

**Figure 6:**
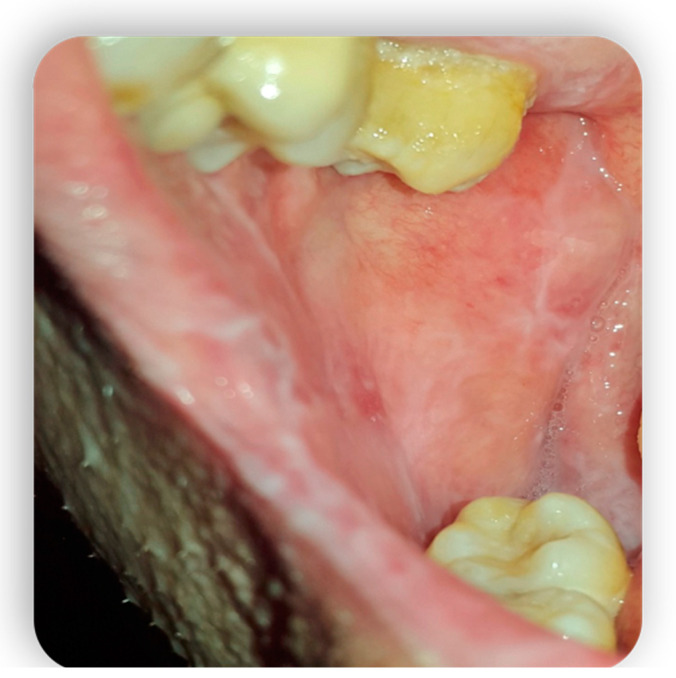
Oral cGvHD manifestation: Labial commissure ulcerations that reduce mouth opening.

Chronic GvHD is the main cause of functional impairment of the oral cavity in patients suffering from GvHD. The oral cavity appears to be the second most frequently involved site in the chronic form in PBSC transplants with a frequency of about 90% (second only to the skin) [34]. The oral cavity is the first involved area in BM transplants (Figure 5 and Figure 6), with a frequency of about 80% [35].

Oral cGvHD lesions can be divided into three groups:

1. Lesions of the oral mucosa
  ○ Localized or diffuse erythema associated with edema or atrophy of the mucosa that reveal the underlying vascular structures [36].
  ○ Lichenoid lesions, white or milky streaks like Wickham’s streaks observed in lichen planus, against which these lesions are compared in differential diagnosis. They usually appear as whitish plaques or spots with hyperplastic mucosa and may have preneoplastic potential [31,37].
  ○ Ulcerations, interruptions in the continuity of the mucosa that emerge when the destroyed epithelium leaves the inflamed connective tissue exposed. Sometimes they can have a pseudomembrane. If they are chronic lesions, they do not heal spontaneously and the reason is that, despite the removal of the traumatic factor that predisposed the formation, there is a low percentage of local growth factors which is also associated with a reduced proliferative capacity of the cells resulting from the disease [31].
  ○ Mucoceles mainly localized on the palate, caused by extravasation of saliva secondary to the sclerotic process of the ductal walls, which obliterates the lumen of the minor salivary glands. The inflammation of the salivary glands, in addition to this stenosis process, is aggravated by the decrease in secretion and the increase in saliva viscosity. The most affected areas are the hard palate and the lower lip [37].
2. Limitation of mouth opening

Chronic inflammation can also cause perioral sclerotic fibrosis, which limits the opening of the mouth due to the overproduction of collagen. CGvHD, as well as scleroderma, can involve any orofacial tissue. The limited opening and the resulting alteration of the oral cavity functions could contribute to the patient’s malnutrition and possible infections. The sclerotic process can also restrict tongue movement and extend to the throat and esophagus, resulting in dysphagia [31].

3. Salivary gland dysfunction

CGvHD, as well as conditioning, can also affect the salivary glands leading to the destruction of the glandular acini with periductal fibrosis. The most frequent consequence is xerostomia with an increase in carious processes and fungal infections. These manifestations are very similar to those of Sjogren’s syndrome, and both are also associated with xerophthalmia. There are three characteristics that distinguish the histopathological alterations of cGvHD in the salivary glands: lymphocytic infiltration in the ducts of the salivary glands, apoptosis of the ductal epithelial cells, and destruction of the tissues of the glandular acini with periductal fibrosis [31].

## 2. Systemic Treatment of Oral Lesions

The treatment of mucocutaneous lesions from acute and chronic GvHD is often a difficult problem for the clinician [38]. In addition to the standard systemic therapies for acute and chronic GvHD, it is possible to use an additional therapy in case of refractory and particularly compromising mucocutaneous manifestations [26]. Much interest has been aroused by the use of Rituximab (anti-CD20 monoclonal antibody) [19,21]. The role played by the cooperation between B and T cells in the activation of immune responses and in the pathogenesis of chronic GvHD could justify the effectiveness of this therapeutic option. Extracorporeal photochemotherapy (ECP), in combination with other immunosuppressive agents, is a very useful option [18]. With ECP, at an extracorporeal level, the blood is treated with a drug activated by UV light that causes direct apoptosis of leukocytes (especially lymphocytes) and is reinfused into the patient. This seems to generate immunological tolerance by interfering with the maturation of the dendritic cells, modulation of cytokine production, and Treg cell expansion. ECP is proposed not only as an alternative therapy but recently also as a first-line therapy for chronic GvHD [16]. The basis of ECP effectiveness is the immunomodulatory effect of the procedure which acts by inhibiting the population of activated donor lymphocytes and altering the interaction between these and the APCs. ECP is an invasive procedure, requiring a dedicated team and several weeks before signs of clinical improvement appear. Some scholars have recently obtained good results using etretinate (synthetic retinoid) [39], in combination with standard therapies, for the treatment of scleroderma resulting from refractory cGvHD. Other authors, especially in consideration of the failure of rescue therapies in multi-treated patients, have experimented with the use of Pentostatin (adenosine deaminase inhibitor), whose effects have already been tested for acute GvHD [21].

## 3. Topical Treatment of Oral Lesions

**Figure 7:**
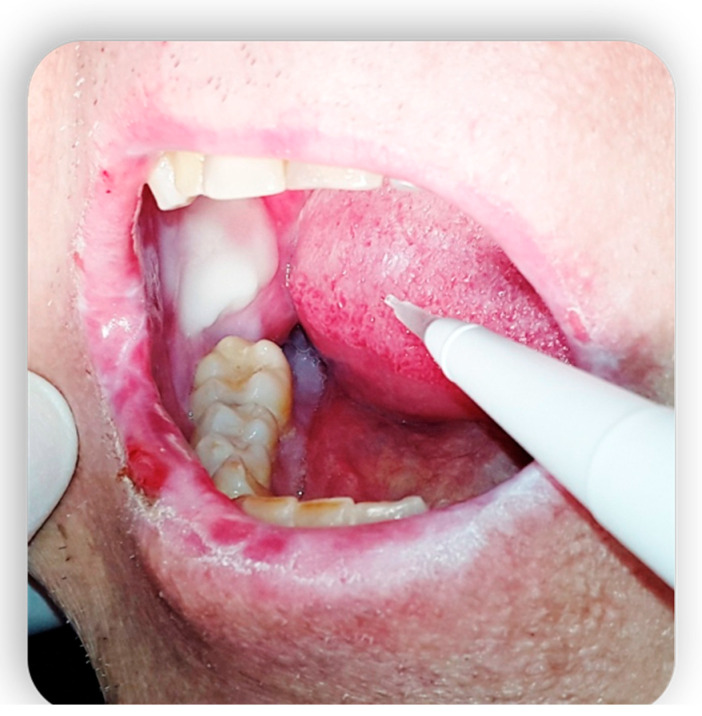
Topical treatment with non-transfusional hemocomponents.

**Figure 8:**
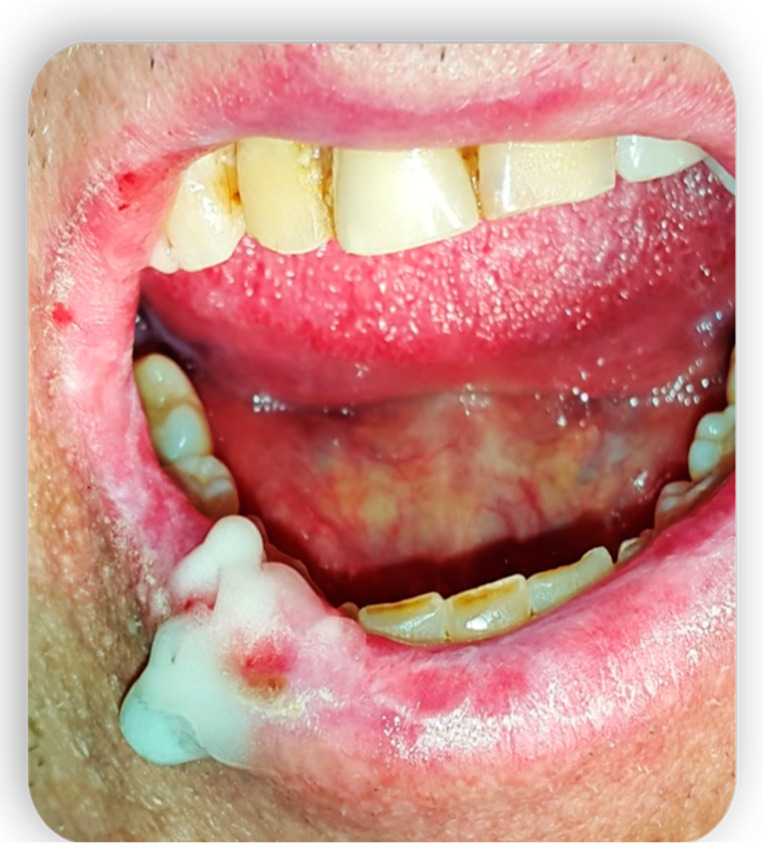
Topical treatment with non-transfusional hemocomponents.

The systemic treatment of the disease consists of immunosuppressive drugs but only in a few cases leads to the healing of oral lesions [38]. These patients could benefit from topical agents, both to focus the action exclusively on the regions of interest and to reduce the state of systemic immunosuppression. In these cases, it is very common to use steroid preparations for topical use such as the combination of fluocinonide and clobetasol, or dexamethasone and betamethasone [40]. The topical application of CSA has also been used successfully in lichenoid manifestations of the mucous membranes and in cases of punctate keratitis. In addition, the application of topical preparations of azathioprine, in association with systemic immunosuppressive therapy, was evaluated in patients with skin ulcerative lesions that are resistant to topical treatment with CSA and cortisone [41]. The results, although obtained from a small number of patients, showed a good response to treatment considering both the extension of the ulcerated areas and pain. Several reports also indicate tacrolimus, already known in the dermatological field for its beneficial effects in atopic dermatitis, as a possible topical agent in the treatment of cutaneous cGVHD [42] (Figure 7 and Figure 8).

## 4. GvHD and Implantology: Case Reports in the Literature

In the current review, there are insufficient references in the literature regarding implant survival and success in patients with GvHD, in any of its forms [43]. Despite this, there are three case reports in the literature that demonstrate the possibility of carrying out an implant treatment plan on a GvHD patient [44].

The first case report is about a clinical case presented by Mahn D.H. of a 46-year-old non-smoker man with a medical history characterized by HSCT-treated non-Hodgkin’s lymphoma, which led to the development of cGvHD 1 year later. Clinical evaluation of the patient revealed remission of the disease, but poor oral hygiene and teeth that were severely compromised by carious disease. Severe gingivitis and gingival recessions were also present. The treatment plan, after evaluating the bone structure, provided for the simultaneous extraction of all the mandibular and maxillary dental elements and the contextual placement of five implants for the lower arch [18]. The treatment was temporarily completed with an immediate upper removable prosthesis and an inferior immediate hybrid prosthesis. After 6 months, the final prostheses were delivered. One year after the completion of the treatment plan, the implant sites were healthy and with stable bone levels. The patient had to maintain good oral hygiene, and no signs of cGvHD were present, apart from oral dryness [45].

In the second case report, Curtis J.W. described the case of a patient with GvHD developed after HSCT. The patient presented with severe caries pathology and sharp enamel projections causing chronic trauma to the tongue and adjacent mucosa. In this case, the teeth were extracted and replaced with removable total prostheses delivered 6 months after the extractions. Only after 2 years, five mandibular implants were inserted to create a complete overdenture delivered 14 months after the insertion of the implants [46].

In the third case report, A. Etebarian et al. [47] presented the case of a patient with cGvHD with a sclerotic phenotype developed after HSCT for the therapeutic treatment of acute myeloid leukemia. The patient is a 35-year-old man who, 1 year after the onset of cGvHD symptoms, has generalized carious pathology with non-recoverable teeth. Therefore, all the elements were extracted, and after healing, there were placed six mandibular endosseous implants. After 6 months, implant success was established according to the criteria of Albrektsson et al., and a fixed prosthesis was made on implants. After 2 years of follow-up, the maxilla was also rehabilitated through the insertion of four endosseous implants and after 6 months the delivery of an overdenture. After 6 years of HSCT transplant, cGvHD resulted in remission and there was a successful oral rehabilitation, significantly increasing the patient’s quality of life [48,49].

Mahn’s case report remains to date the only one that describes an implant success in a patient with GvHD in which dental extractions and simultaneous insertion of mandibular implants were performed [41,50].

In conclusion, what are the keys to successful case management? Attention to detail during the diagnostic, surgical, and restorative phases, management of patient expectations, and compliance with patient instructions.

Extremely important, then, is patient compliance; only then can predictable results be achieved and complications averted.

## 5. Conclusions

The mechanisms underlying the development of GvHD are becoming increasingly clear but enormous progress still needs to be made in this regard. Therapeutic approaches can be established only through a full understanding of the disease. Oral manifestations of the disease, especially in its chronic form, are highly frequent and their management is not easy for the dentist. The mucosa of the post-HSCT patient loses its ability to renew itself due to the impact of the treatments to which it is subjected. Treatment options, in the current review, are numerous but almost none of them are yet supported by randomized controlled trials (RCTs) demonstrating their benefits. Therefore, there is a need to develop new therapeutic techniques for topical use in addition to the traditional use of immunosuppressive drugs, such as platelet gels, and the use of new and interesting proposals that have the potential to become first-line treatments in dentistry for the resolution of oral lesions. Implant therapy in the GvHD patient also needs further study as it could become a very valid opportunity for rehabilitation of the oral cavity and significantly increase the quality of life of patients suffering from this pathology. In this context, the synergy between the dentist and other relevant medical figures is crucial to achieve predictable results. The relationship between the dentist and the specialist is bidirectional, as it is important that the dentist release a dental clearance before the patient can start HSCT. For this reason, as previously stated, it is essential to perform all the necessary treatments for the elimination of traumatic and inflammatory factors. When the dentist is faced with such a situation, it is good that he or she knows the characteristics and mechanisms of this pathology in order to be able to treat the oral lesions.

## Figures and Tables

**Table 1:** Billingham’s criteria.

Pathology	Pathological State	Treatment Options
Carious pathology	▪ Mild or moderate ▪ Severe	Restorative (if sufficient timing or no treatment), Pulpectomy
Apical paradentitis	▪ Present symptomatology ▪ Absent symptomatology	Extraction or root canal therapy (depending on available time frame) Extraction or root canal therapy if radiotransparency > 5 mm No treatment for radiotransparency < 5 mm
Periodontal disease	▪ Present ▪ Absent, with PPD > 8 mm and severe mobility ▪ Absent, with PPD < 8 mm and mild or moderate mobility	Extraction Extraction Renew dental hygiene instructions and perform scaling maneuvers
Partially erupted third molars	▪ Eruption difficulties ▪ No eruption difficulties	Extraction No treatment

## Data Availability

Not applicable.
